# Biological Effect of *Streptococcus pyogenes*-Released Extracellular Vesicles on Human Monocytic Cells, Induction of Cytotoxicity, and Inflammatory Response

**DOI:** 10.3389/fcimb.2021.711144

**Published:** 2021-07-19

**Authors:** Kazunori Murase, Chihiro Aikawa, Takashi Nozawa, Ayako Nakatake, Kuniyo Sakamoto, Taisei Kikuchi, Ichiro Nakagawa

**Affiliations:** ^1^ Department of Microbiology, Graduate School of Medicine, Kyoto University, Kyoto, Japan; ^2^ HTLV-1/ATL Research Facility, Faculty of Medicine, University of Miyazaki, Miyazaki, Japan; ^3^ Department of Infectious Diseases, Faculty of Medicine, University of Miyazaki, Miyazaki, Japan

**Keywords:** extracellular vesicles, *Streptococcus pyogenes*, pathogenicity, cytotoxicity, inflammatory response

## Abstract

Most bacteria naturally release spherical lipid-bilayered extracellular vesicles (EVs) containing proteins, nucleic acids, and virulence-related molecules, thus contributing to diverse biological functions including transport of virulence factors. The group A streptococcus, *Streptococcus pyogenes* (GAS), a major human pathogen, also releases EVs; however, it remains unclear how GAS EVs interact physiologically and pathologically with host cells, and what the differences are between invasive and non-invasive strains. The proteome profile in this study revealed that GAS EVs enclosed many virulence-related proteins such as streptolysin O and NAD-glycohydrolase, facilitating their pathogenicity, and invasive GAS EVs were more abundant than non-invasive counterparts. In terms of biological effects, invasive GAS EVs showed *slo*-dependent cytotoxic activity and the induction of cytokine expression, contributing to GAS pathogenicity directly. Although non-invasive GAS EVs did not show cytotoxic activity, they may be utilized as a means to prevent antibacterial mechanisms such as autophagy, leading to enhancement of their own survival in the intracellular environment after the infection. These results suggest that invasive and non-invasive GAS EVs play different roles in GAS infection strategy and pathogenicity. Our findings also indicate that EVs could be a key factor for GAS pathogenicity in GAS-host interactions.

## Introduction

Most bacteria naturally release spherical lipid-bilayered extracellular vesicles (EVs) with sizes ranging from 20 to 400 nm in diameter (Toyofuku et al., 2019). In gram-negative bacteria, EVs are produced by two main routes: blebbing of the outer membrane and/or explosive cell lysis. The formation of these EVs is induced by environmental triggers such as iron limitation, antibiotics, and peptidoglycan-degrading enzymes (Schwechheimer and Kuehn, 2015; Jan, 2017; Toyofuku et al., 2019). In gram-positive bacteria, mechanisms of EV production have not been well elucidated compared with gram-negative bacteria. However, it is likely that gram-positive bacteria produce EVs by protruding the cytoplasmic membrane and release them through holes in the thick peptidoglycan formed by endolysins (Brown et al., 2015; Nagakubo et al., 2019). Furthermore, both gram-negative and -positive bacterial EVs enter host cells by several mechanisms, including membrane fusion, lipid rafts, and clathrin- or caveolin-mediated endocytosis pathways (O’donoghue and Krachler, 2016). The nano-sized EVs enclose numerous bacteria-derived products, including proteins, DNA, RNA, and virulence-related molecules, contributing to diverse biological functions such as transport of virulence factors and cellular metabolites, horizontal gene transfer, and cell-to-cell communications (Kulp and Kuehn, 2010; Toyofuku et al., 2019).

Recent studies have demonstrated that the clinically important gram-positive bacteria, such as *Staphylococcus aureus*, *Streptococcus pneumoniae*, and Bacillus anthracis, also produce and secrete EVs with a large diversity of cargo components (Liu et al., 2018). In pneumococci such as *S. pneumoniae*, EVs are released in the host and involved in avoidance of complement deposition and phagocytosis-mediated killing, thereby possibly contributing to the symptoms of pneumococcal infections (Codemo et al., 2018). In *S. aureus*, EVs are associated with the induction of atopic dermatitis-like inflammation and apoptosis of Hep-2 cells (Gurung et al., 2011; Hong et al., 2011). Thus, EVs are strongly involved in the interaction between pathogens and the host, and could be an important factor for pathogenicity during bacterial infection.

*Streptococcus pyogenes* (group A Streptococcus, GAS) is a human pathogen that causes streptococcal pharyngitis, skin and soft tissue infections, and life-threatening conditions such as streptococcal toxic shock syndrome (STSS) (Cole et al., 2011). Globally, GAS is one of the most predominant infectious causes of human morbidity and mortality, responsible for over 600 million cases and 500,000 deaths every year (Efstratiou and Lamagni, 2016). GAS strains are classified based on serological differences in the M protein, an anti-phagocytic cell-surface molecule. Although more than 100 distinct M types have been identified to date, particular M types, including M1, M3, M12, and M89, which are associated with invasive GAS diseases, have been frequently isolated (Gherardi et al., 2018; Musser, 2020).

This bacterium has a single chromosome of roughly 1.9 Mb that encodes a wide range of virulence determinants with diverse biological activities involved in a complex series of physiological processes (Nakagawa et al., 2003; Davies et al., 2019). These virulence determinants play appropriate temporal roles in specific diseases, leading to a variety of invasive conditions that are associated with high morbidity and mortality. The CovRS system, one of the best characterized two-component regulatory systems in GAS, is involved in the regulation of around 15% of the GAS genome, and mutations of *covRS* results in the upregulation of many GAS virulence factor genes, including *sda1*, *ska*, *slo*, and *hasABC* (Graham et al., 2002). Thus, the *covRS* mutant form is associated with GAS pathogenicity and may also lead to the emergence of invasive GAS strains. However, mutations in the CovRS system were not necessarily linked to lethal infection by strains with invasive potential (Tse et al., 2012), implying alternative mechanisms and/or factors for virulence determinants in GAS.

It has been reported that GAS also actively produces EVs, and that their production is increased in the *covRS* mutant form and upon treatment with sublethal penicillin or antimicrobial peptide LL-37 (Biagini et al., 2015; Resch et al., 2016; Uhlmann et al., 2016). However, there is no clarity on differences between invasive and non-invasive strains in terms of EV production, or regarding how GAS EVs physiologically or pathologically affect host cells.

In this study, we performed comprehensive proteomic analysis of EVs from two representative GAS strains: an invasive strain showing the high-virulence phenotype, SSI-1 (serotype M3) isolate that caused STSS; and a non-invasive strain most widely used in studies of bacteria-host cell interactions, JRS4 (serotype M6) isolate that caused rheumatic fever. While JRS4 causes non-invasive disease, this strain is known to be capable of cell invasion during infection. Our study revealed that GAS EVs enclose many virulence-related proteins, but their content and abundance is different between invasive and non-invasive strains. Furthermore, GAS EVs were incorporated into host cells *via* the endocytic pathway and induced cytotoxic activity and inflammatory responses. This study provides the basis for further studies on the impact of EVs on the pathogenicity of GAS and may provide clues to understanding the diverse symptoms induced by GAS infection.

## Experimental Procedures

### Bacterial Strains and Culture Conditions

Two *S. pyogenes* strains SSI-1 (a serotype M3 isolate that caused STSS) and JRS4 (a serotype M6 derivative of D471 isolate that caused rheumatic fever) were used in this study (Scott et al., 1986; Nakagawa et al., 2003). For routine bacterial cultivation, bacterial cells were grown at 37°C in Todd-Hewitt broth supplemented with 0.2% yeast extract (THY) without shaking.

### Construction of *Slo* Mutant Stains

To delete the *slo* gene, we used a two-step allele exchange method with the thermo-sensitive pSET4s vector, as described previously (Roobthaisong et al., 2017). Briefly, the 5′ and 3′ flanking regions (each 800 bp) of *slo* were amplified from GAS SSI-1 and JRS4 genomic DNA using the primer pairs listed in **Supplementary Table 1**. The resultant amplicons were joined to *Sma*I-digested pSET4s by Gibson Assembly (New England BioLabs). The pSET4s carrying the gene deletion allele was transformed into *E. coli* strain DH10B and grown on LB agar plates supplemented with 100 µg ml-1 spectinomycin (Nacalai Tesque). The resultant clones were confirmed to contain the deletion allele by colony polymerase chain reaction (PCR). The purified plasmid was introduced into wild-type GAS cells, and the cells were then incubated on THY agar plates (containing 100 µg ml-1 spectinomycin) at a permissive temperature of 28°C. To generate chromosomal single-crossover mutants, selected colonies were further grown on THY agar plates containing spectinomycin at 37°C. The single-crossover mutants were then sub-cultured to a permissive temperature of 28°C without spectinomycin for induction of the second-crossover event. Spectinomycin-sensitive colonies were screened for the gene deletion or reverted to the wild-type genotype by colony PCR.

### Preparation of Extracellular Vesicles

Bacterial cells were grown to an OD600 of 0.8-1.0, at 37°C in THY media, either supplemented or not, with 4 µM 1,1-dioctadecyl-3,3,3,3-tetramethylindocarbocyanine (DiI) stain solution (ThermoFisher), and centrifuged at 7000 × g for 10 min at 4°C. We confirmed that cells and the extracted EVs were stained with DiI (**Supplementary Figure 1**) without any inhibition during growth. The culture supernatants were filtered (pore size, 0.45 µm) to obtain cell-free supernatants. The filtered supernatants were further ultra-centrifuged at 150,000 × g for 3 h at 4°C, and the pellets were washed with 1xPBS three times. The washed pellets were resuspended in 20 mM Tris-HCl (pH 7.8) and stored at -80°C until use. The protein concentration of EVs was determined using a BCA Protein Assay Kit (ThermoFisher) according to the manufacturer’ s instructions.

### Observation and Quantification of EVs

Five microliters of the EV solution were dropped onto a collodion-coated copper grid and blotted using filter paper after 5 min. The grid was then stained for 45 s by inverting onto a drop of 2% uranyl acetate solution and blotted using filter paper. The EVs were visualized by transmission electron microscopy (TEM; HT7700, HITACHI) operating at 80 kV. Size distribution and quantification of EVs were performed by nanoparticle tracking analysis using a NanoSight NS300 (Malvern, UK) according to the manufacturer’s instructions. We further calculated the EV production amounts based on the total cell numbers in colony forming units (CFUs)/ml. This analysis was repeated at least twice for each sample.

### Proteome Analysis

Proteins from EVs were trypsinized in buffer (1 M urea and 50 mM NH4HCO3) at 37°C for 20 h with mixing. The digested solution was acidified with 0.8% formic acid (final concentration) and centrifuged at 15,000 × g for 5 min. The supernatant was collected, and an equal volume of ethyl acetate was added. The obtained lower aqueous phase was dried, and the resultant pellet was resuspended in 0.1% formic acid. The resultant peptide solution at a concentration of 500 ng/µL was then subjected to LC-MS/MS analysis.

Mass spectrometry was performed on the Q-Exactive spectrometer (Thermo Fisher Scientific, San Jose, CA, USA). Chromatography was performed using the UltiMate3000 RSLCnano (Thermo Scientific) system (flow rate, 300 μL/min) on an EASY-Spray column (25 cm × 75 μm ID, PepMap C18, 2 µm; Thermo Scientific) with a 70 min gradient. Solvent A was water/0.1% formic acid, and solvent B was acetonitrile/0.1% formic acid; peptides were eluted using a gradient from 1% to 35% solvent B. A precursor scan of intact peptides was measured in the orbitrap by scanning from m/z 380–1500 with a resolution of 70,000, from which the 10 most intense multiply-charged precursors were selected for higher-energy collisional dissociation analysis in the C-trap under the following conditions: a resolution of 17500 and an isolation window of 1.6 m/z. The normalized collision energy was set at 27.0. Automatic gain control targets were 3e6 and 1e5 ions for orbitrap scans (maximum injection time 60 ms) and MS/MS scans (maximum injection time 200 ms), respectively. Dynamic exclusion for 20 s was used to reduce repeated analyses on the same components.

Protein identification using the MS/MS data was performed using Proteome Discoverer software (ver.2.2, Thermo Fisher Scientific) using the Sequest HT search algorithms. In the Sequest HT search, whole SSI-1 (1,800) and JRS4 (1,663) protein sequences were used for protein identification. Proteome data can be found in jPOSTrepo (Japan proteome standard repository) under the ID: JPST000954 and/or http://www.proteomexchange.org/, under the ID: PXD026040.

### COG/KO Analysis and GO Enrichment Analysis

Clusters of orthologous groups (COGs) were analyzed according to a previously described method (Her and Wu, 2018). Briefly, COGs were predicted by searching the amino acid sequences of the genes against COG hidden Markov models downloaded from the eggNOG 4.5 database (Huerta-Cepas et al., 2016) by using HMMER3 (Eddy, 2011) with an e-value cutoff of 1e-5. Protein functions were further assigned using the BlastKOALA tool *via* the KEGG database (Kanehisa et al., 2016). KEGG Orthology (KO) terms were divided into 13 supercategories including “Genetic Information Processing,” “Metabolism,” “Cellular Processes,” and “Environmental Information Processing,” based on the KEGG hierarchy classification.

GO enrichment analysis was performed as follows. Briefly, we performed functional annotation on the identified proteins using several bioinformatics tools. The protein sequences were annotated by Protein Basic Local Alignment Search Tool (BLASTP) searches against the non-redundant (nr) protein database at the National Center for Biotechnology Information (https://www.ncbi.nlm.nih.gov). InterProScan was also used to search against the InterPro protein family database, which included PROSITE, PRINTS, Pfam, ProDom, the Simple Modular Architecture Research Tool (SMART), TIGRFAMs, PIR SuperFamily (PIRSF), SUPERFAMILY, signal peptide (SignalP), and transmembrane helices hidden Markov models (TM-HMM). The search results were then loaded into Blast2GO v.5.2.5 to annotate the sequences with GO terms. GO enrichment analyses were performed with the whole SSI-1 (1,800) and JRS4 (1,663) proteins as the references using Fisher’s exact test with the p-value implemented in Blast2GO software. Enrichment scores were calculated using the log10-transformed p-value (P < 0.01).

### Cell Culture Conditions

Human monocytic leukemia (THP-1) cells were purchased from the ATCC. The cells were grown at 37°C in a 5% atmosphere in complete medium consisting of RPMI1640 (Nacalai Tesque), supplemented with 10% fetal bovine serum (FBS; Gibco) and 50 ng ml-1 gentamycin (Nacalai Tesque). The cells were sub-cultured twice a week with 1 × 10^6^ viable cells ml-1 and incubated at 37°C in a 5% CO2 atmosphere. For the infection assay, THP-1 cells were treated with 50 ng ml-1 phorbol-12-myristate-13-acetate (PMA) in order to differentiate into adherent macrophage-like cells (dTHP-1). Then, the cells were plated at 1 × 10^5^ cells per well in 24-well flat-bottom culture plates and incubated for 72 h at 37°C in a 5% atmosphere. The cells were washed with PBS three times prior to media replacement and then further incubated for 24 h.

### Uptake Assay of EVs Into dTHP-1 Cells and the Microscopy Observation

A total 10 µg of DiI-labeled EVs were added to dTHP-1 cells (1 × 10^5^ cells/well in 24-well plates) and incubated for 2 h at 37°C with 5% CO2. The same procedure was performed with incubation at 4°C for 2 h to assess the effect of temperature on EV uptake.

a) Confocal laser scanning microscopy observation

Cells were washed twice with PBS and fixed with 4% paraformaldehyde in phosphate-buffered saline (PBS) for 15 min. After washing twice with PBS, cellular DNA was stained with 4,6-diamidino-2-phenylindole (DAPI; Dojindo) in PBS. DAPI was visualized with a 405-nm laser and DiI with a 561-nm laser. Fluorescence confocal microscopy images were acquired using an FV1000 laser-scanning microscope (Olympus).

b) Flow cytometry analysis

After incubation, the cells were washed three times with HBSS buffer to remove any residual EVs and treated with Accutase to detach and dissociate the cells. Cellular suspensions were collected in FACS tubes and diluted with 2% FBS in HBSS until measurement with a Cell Analyzer EC800 (Sony Corp., Tokyo, Japan). The procedure was also performed after incubation at 4°C for 2 h. As mentioned before, a threshold of 10,000 live cells was set for analysis from FSC *vs*. SSC gating. DiI-positive cells (PE channel) after uptake of DiI-labeled EVs were determined, as compared with cells treated with 4 µM DiI solution only (negative control), using FlowJo 10.6.1 software (FlowJo LLC, USA).

### Cytotoxicity and Cytokine Activity Assay

A total of 10 µg of EV solution was treated with dTHP-1 cells, which were seeded at 1 × 10^5^ cells/well in 24-well plates and differentiated for 72 h. After incubation for 4 h in RPMI1640 supplemented with 5% FBS at 37°C in 5% CO2 atmosphere, the supernatants were used for cytotoxicity and cytokine activity assays.

The cytotoxicity assay was performed using a lactate dehydrogenase (LDH) Cytotoxicity Assay Kit (Nacalai Tesque) according to the manufacturer’s instructions, and the release of LDH was measured by the absorbance at 450 nm by using an iMark microplate reader (Bio-Rad). For the assessment of cytokine activity, interleukin (IL)-8, tumor necrosis factor (TNF)-α, and IL-1beta were quantified using a human IL-8/TNF-α/IL-1beta ELISA kit (Proteintech) according to the manufacturer’s instructions.

### RNA Preparation, cDNA Synthesis, and Quantitative Real-Time (RT) PCR

Total RNA from dTHP-1 cells treated with 10 µg EV solution or PBS was extracted and purified using Quick-RNA Miniprep (Zymo Research) according to the manufacturer’s instructions and quantified using an ND-1000 spectrophotometer (NanoDrop). Purified RNA (500 ng) was immediately reverse transcribed using the PrimeScript II 1st strand cDNA Synthesis Kit (Takara) according to the manufacturer’s instructions, and the resultant cDNA was used for real-time PCR assay. The cDNA samples were stored at -20°C until downstream analysis.

Real-time PCR assays were carried out in a CFX Connect Real-Time PCR Detection System (Bio-Rad) using SsoAdvanced Universal SYBR Green Supermix (Bio-Rad), following the manufacturer’s instructions. Prior to the real-time PCR assay, we preliminarily carried out gradient PCR with primer pairs (**Supplementary Table 1**) to optimize the PCR conditions. PCR amplification was performed in a reaction mixture that contained template cDNA, each primer pair, and 1x SYBR Green Supermix adjusted to a final volume of 20 µL under the following conditions: denaturation for 3 min at 95°C; 40 cycles of 10 s at 95°C and 30 s at 56°C. Finally, dissociation curve analysis was performed to check for non-specific amplification and/or primer-dimer formation.

Standard curves were estimated for every candidate reference gene to determine the PCR amplification efficiency and the regression coefficient (R2) of each pair of primers. Ten-fold serial dilutions of THP-1 genomic DNA were used in real-time PCR reactions, and the five-point standard curves were generated by plotting the average CT numbers versus the logarithm of the amount of template DNA. The PCR amplification efficiency (E) of each primer pair was calculated according to the formula: E (%) = [10(-1/slope) -1] × 100 (Wong and Medrano, 2005). The efficiency of each reference gene was considered in all the statistical analyses.

### Statistical Analysis

Values, including plotted values, represent the mean ± standard error of the mean (SEM). Data were tested using the two-tailed Student’s t-test, and P < 0.05, was considered significant (*P < 0.05, **P < 0.01, ***P < 0.001, ****P < 0.0001; ns, not significant).

## Results

### *S. pyogenes* Releases Extracellular Vesicles

**Figure 1:**
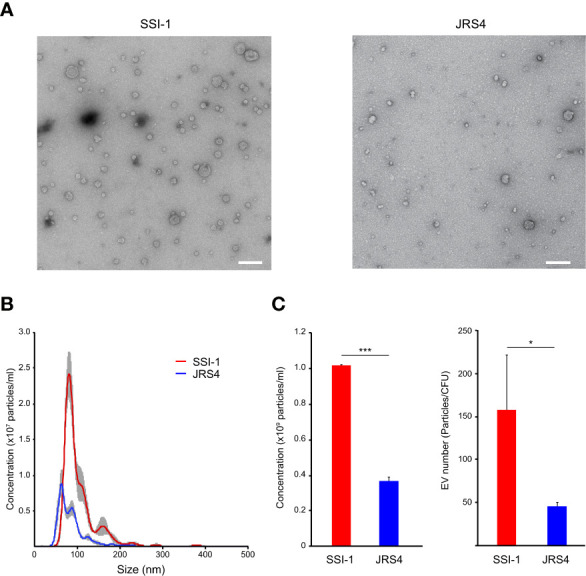
Observation and quantification of EVs released from *S. pyogenes* str. SSI-1 and JRS4. **(A)** Extracted EVs were negatively stained and visualized by TEM at on original magnification of 30,000. Scale bars, 200 nm. **(B)** Quantification and size distribution of *S. pyogenes* EVs were analyzed using NanoSight. **(C)** Concentrations (particles/ml) and EV numbers (particles/CFU) were calculated from the NanoSight data and are shown in the left-hand and right-hand panels, respectively. All experiments were performed in triplicate. Data in B represent the mean ± SEM of independent experiments. P-value calculated by two-tailed Student's t-test. *P < 0.05, ***P < 0.001.

We collected and observed EVs from two *S. pyogene*s strains: the invasive SSI-1 and the non-invasive JRS4 (**Figure 1A**). The size distribution of EVs of both strains was in the range of 40–250 nm, but SSI-1 EVs (101.7 ± 1.1 nm) were 1.31 times bigger than JRS4 EVs (84.2 ± 0.4 nm) (P<0.01; **Figure 1B**). Notably, the EV amounts in SSI-1 and JRS4 were 1.01×10^9^ and 3.62×10^8^ particles/ml, respectively, and the quantification profile demonstrated that SSI-1 produced 2.79 times more EVs than JRS4 (P<0.05; left-hand panel of **Figure 1C**). Furthermore, the EV numbers (particles/CFU) were 158 (± 76.3) and 45 (± 4.7) in SSI-1 and JRS4, respectively (right-hand panel of **Figure 1C**).

### GAS-Derived EVs Enclose and Enrich Many Virulence-Related Proteins

**Figure 2:**
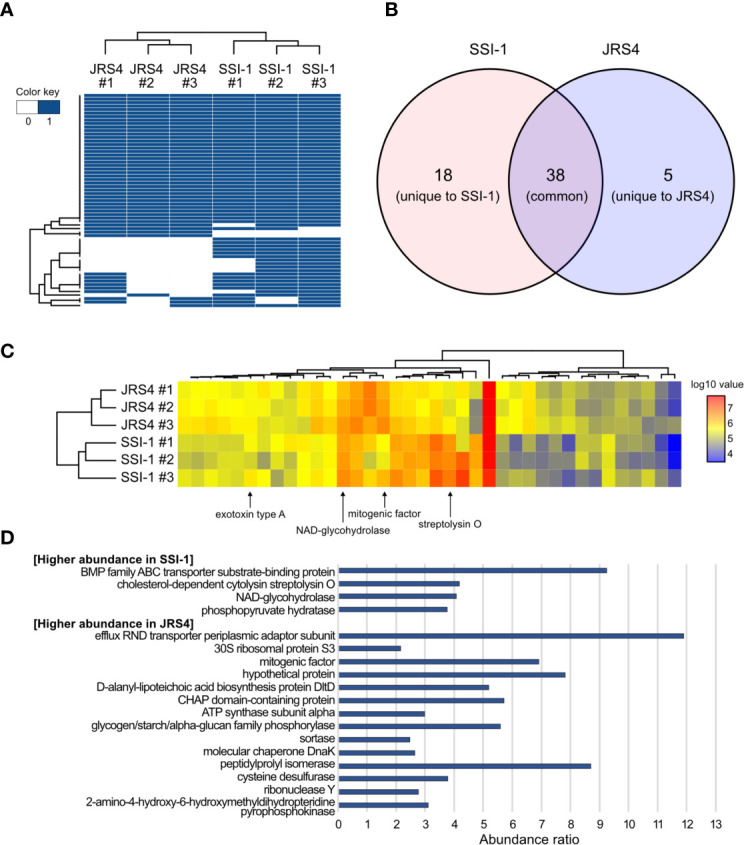
Comparison of proteome profiles of EVs from *S. pyogenes* str. SSI-1 and JRS4. **(A)** Heatmap representation of clustering according to presence and absence of proteins in EV from SSI-1 and JRS4, with biological triplicates. **(B)** Venn diagram of EVs-derived proteins identified in SSI-1 and JRS4. **(C)** Heatmap representation of clustering according to log10-transformed abundance values of common proteins identified in two strains. Arrows indicate well-known virulence factors in GAS. **(D)** Abundance ratio significantly higher (> 2.0) in SSI-1 or JRS4 that are shown in bar-chart. Heatmap generation was implemented in R by using heatmap.2 from ggplots package.

Mass spectrometry analysis of EVs from SSI-1 and JRS4 strains identified 56 and 43 proteins, respectively (**Figure 2A**). We found that the GAS EVs contained various functional proteins, such as virulence-related proteins (streptolysin O, NAD-glycohydrolase, C5a peptidase, exotoxin type A/C, and streptokinase); cell surface-related proteins (D-alanyl-lipoteichoic acid biosynthesis protein, LPXTG cell wall anchor domain-containing protein, efflux RND transporter periplasmic adaptor subunit protein, and ABC transporter-related protein); enzymatic proteins including metabolic pathway proteins (ribonuclease Y, endopeptidase, hydrolase, aldehyde dehydrogenase, and pyruvate kinase); and ribosomal proteins (**Supplementary Table S2**). Among them, 38 proteins were common (ortholog proteins) in both strains, and 18 and five proteins were unique to SSI-1 and JRS4 strains, respectively (**Figure 2B**). When comparing proteins commonly identified in both strains, four and 15 proteins were greater than two-fold more abundant in SSI-1 and JRS4 strains, respectively (**Figures 2C, D**). Furthermore, two major virulence factors—streptolysin O and NAD-glycohydrolase—were more abundant in SSI-1 (approximately four-fold) (**Figure 2D**). Conversely, surface antigen-related mitogenic factor and several enzymatic proteins remained more abundant in JRS4 (approximately two- to five-fold). Conversely, other virulence factors—C5a peptidase and exotoxin type C/streptokinase—were specifically present only in SSI-1 EVs and JRS4 EVs, respectively.

**Figure 3:**
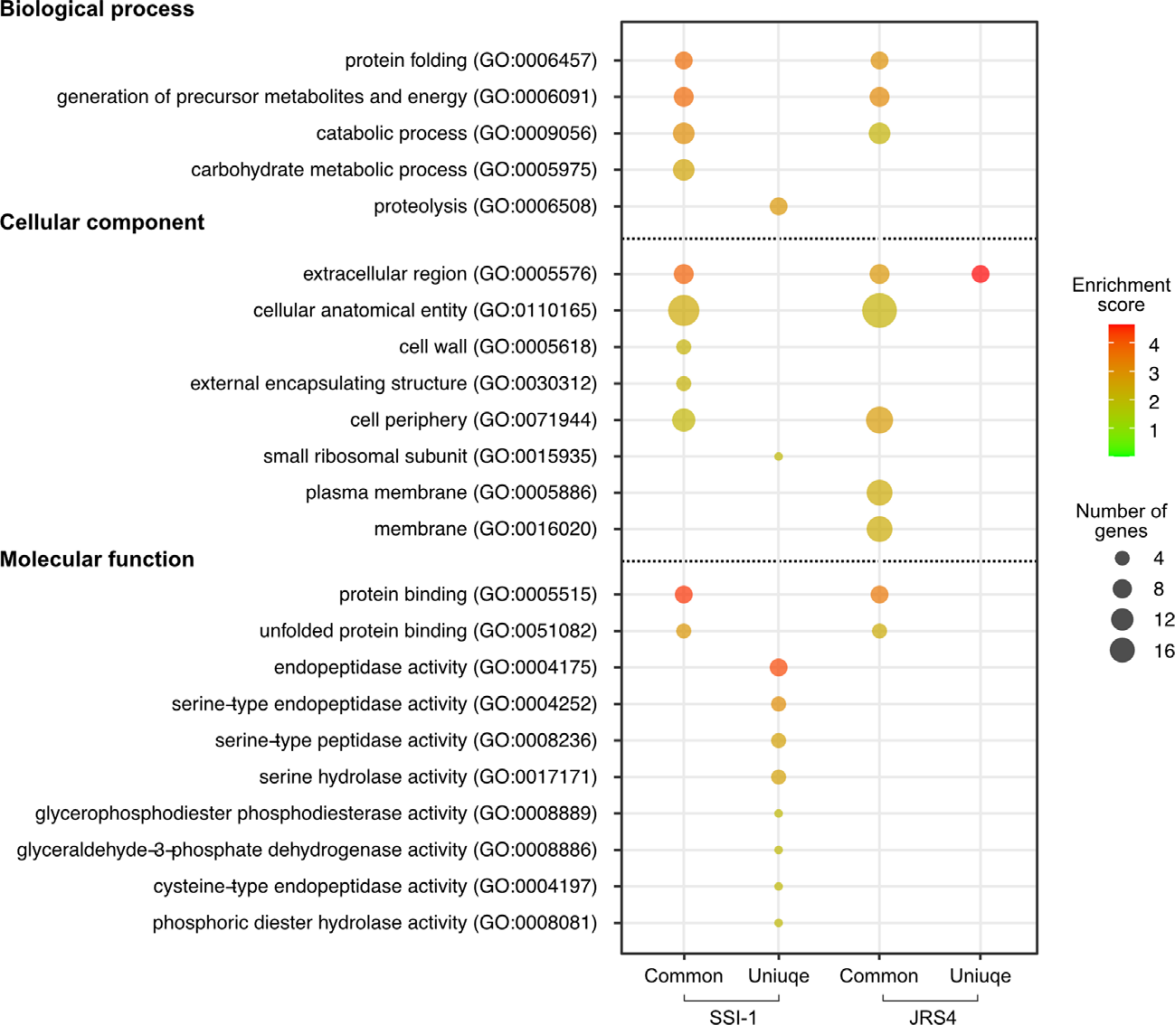
GO enrichment analysis of proteins identified in EVs from SSI-1 and JRS4. GO enrichment analyses were performed with the whole SSI-1 and JRS4 proteins as references by using Fisher’s exact test with p-value. Enrichment scores are calculated by -log10-transformed p-value (P<0.01), and the scores and the number of genes corresponding to each GO term is represented by color key and circle size, respectively. Plot generation was implemented in R. The numbers shown in the parentheses indicate GO number corresponding to the GO terms.

Gene ontology (GO) functional enrichment analysis highlighted significant enrichment (Bonferroni-corrected P values, P<0.01). Common proteins of the two strains showed similar enrichment patterns in all three categories (biological process, cellular components, and molecular function), except for carbohydrate metabolic process (GO:0005975), cell wall (GO:0005618), and external encapsulating structure (GO:0030312), which were enriched in SSI-1, and plasma membrane (GO:0005886), and membrane (GO:0016020), which were enriched in JRS4 (**Figure 3** and **Supplementary Table S3**). However, the enrichment patterns of unique proteins in each strain were completely different. While only the extracellular region (GO:0005576) was enriched in JRS4, proteolysis (GO:0006508) and eight GO categories related to enzymatic activity including peptidase were enriched in SSI-1. These results suggest that EVs potentially have different biological activity in the two strains even though their production mechanism seems to be the same. Clusters of orthologous groups (COG) functional analysis revealed that proteins shared by the two strains were enriched in categories “D” (cell cycle control, cell division, chromosome partitioning), “E” (amino acid transport and metabolism) and “M” (cell wall/membrane/envelope biogenesis). The 18 SSI-1-specific proteins were classified into “F” (nucleotide transport and metabolism) and “I” (lipid transport and metabolism) in addition to D, E and M. In Kyoto encyclopedia of genes and genomes (KEGG) analysis, EV proteins were assigned to 13 KEGG orthology (KO) categories, and four were predominant KO categories in the common protein group (environmental information processing, carbohydrate metabolism, genetic information processing, and metabolism). SSI-1- and JRS4-specific proteins were assigned to eight and two categories, respectively, and the “lipid metabolism” category was found only in the SSI-1-specific group.

### Uptake of EVs Is Dependent on The Endocytic Pathway

**Figure 4:**
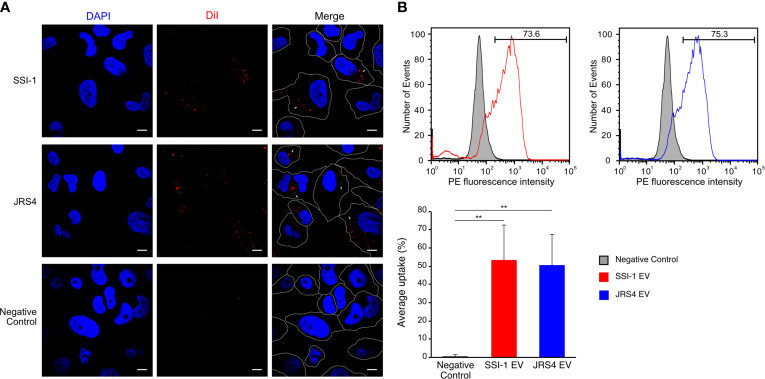
Uptake of DiI-labeled EVs and the efficiency in dTHP-1 cells. **(A)** The dTHP-1 cells were co-incubated with 10 µg of DiI-labled EVs (red) solution for 2 h at 37°C and 4°C (shown in **Supplementary Figure 3**) and observed by confocal fluorescence microscopy. Cellular DNA was stained with DAPI (blue). Scale bar, 5 µm. Dot lines indicate the border of individual cells. **(B)** Flow cytometric analysis of the fluorescence transferred by EVs into dTHP-1 cells. The staining of directly labelled cells and the negative control (cells treated with 4 µM DiI solution) are shown as overlays. The PE fluorescence intensity was measured after 2 h incubation period. Average percentage uptake of DiI-labelled EVs into dTHP-1 cells was represented by bar chart with standard deviation of independent experiments. P-values calculated by two-tailed Student’s t-test. **P < 0.01.

It has been reported that bacterial EVs enter into host cells and modify host cell physiology *via* the activity of EV-enclosed biomolecules (O’donoghue and Krachler, 2016). We first observed such interactions using purified GAS-derived EVs and THP-1 cells. Confocal laser scanning microscopy images showed the internalization of EVs within the cytoplasm of dTHP-1 cells after a 2h incubation period, as detected by the appearance of red dots of DiI-labeled EVs (**Figure 4A**). In contrast, no uptake of EV particles was observed in THP-1 cells cultured at 4°C (**Supplementary Figure 3**). To quantify the uptake of EVs, we performed a flow cytometry assay with dTHP-1 cells either treated with DiI-labeled EVs or DiI solution only. The flow cytometry profile showed a different peak with higher fluorescence intensity in dTHP-1 cells treated with SSI-1 or JRS4 EVs, compared with the control treated with DiI solution only (**Figure 4B**). The uptake ratios in cells exposed to SSI-1 and JRS4 EVs were 53.1% and 50.3%, respectively. These results indicate that GAS-derived EVs are incorporated into dTHP-1 cells *via* the endocytic pathway.

### GAS EVs Induce Inflammatory Responses and *Slo*-Dependent Cytotoxic Activity in dTHP-1 Cells

**Figure 5:**
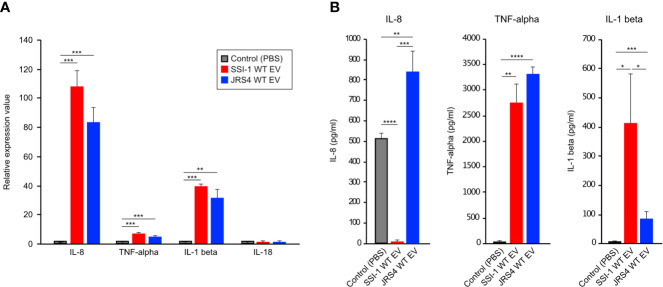
Induction of cytokine expression in dTHP-1 cells treated with GAS EVs. 10 µg of EVs solution was treated with dTHP-1 cells and incubated for 4 h at 37°C. Total RNA extracted from the cells were used for transcriptional level of IL-8, TNF-α, IL-1 beta, and IL-18 by using real-time PCR assay **(A)**, and the supernatants were directly used for ELISA assays of IL-8, TNF-α, IL-1 beta **(B)**. PBS-treated dTHP-1 cells were used as a control for the experiments, and all experiments were performed in triplicate. Data in this analysis represent the mean ± SEM of independent experiments. P-values calculated by two-tailed Student’s t-test. *P<0.05, **P < 0.01, ***P < 0.001, ****P < 0.0001.

To better understand the effect of EV uptake on host immune cells, we first assessed the expression of four key cytokines (IL-8, TNF-α, IL-1β, and IL-18) in dTHP-1 cells at the transcriptional level. In cells incubated at 37°C for 4 h, EVs from both SSI-1 and JRS4 markedly stimulated IL-8 expression (107.5 and 84.4-fold, respectively) (**Figure 5A**). Two cytokines, TNF-α and IL-1β, also showed significantly higher expression in both SSI-1- and JRS4-treated samples compared with controls, whereas no significant changes were seen in IL-18 expression (**Figure 5A**). No cytokines were induced at the transcriptional level in dTHP-1 cells incubated at 4°C when co-cultured with EVs (**Supplementary Figure 4**). These results suggest that cytokine expression is upregulated by uptake of EVs.

We further investigated cytokine production of IL-8, TNF-α, and IL-1β using ELISA (**Figure 5B**). The production of two cytokines—TNF-α and IL-1β—was consistent with their transcriptional profile and was induced in cells that were co-incubated with EVs from SSI-1 or JRS4. However, there was no IL-8 production in the cells treated with SSI-1 EVs, even though the transcriptional expression was strongly upregulated.

**Figure 6:**
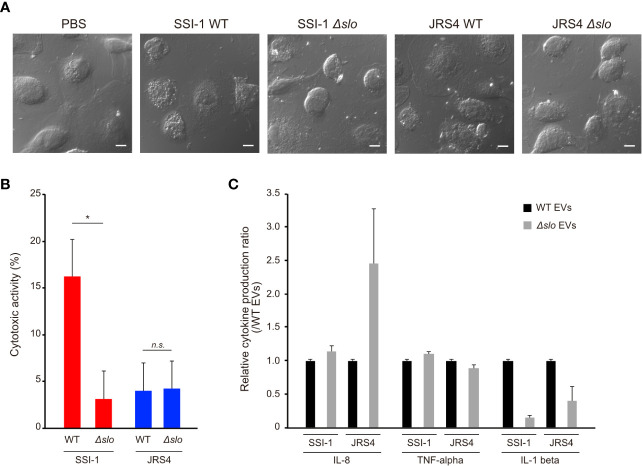
Induction of cytotoxic activity and cytokine expression in dTHP-1 cells by EVs-mediated SLO. The dTHP-1 cells were co-incubated with 10 µg of DiI-labled EVs isolated from two GAS wild-type (WT) and the *slo* mutants (∆*slo*) and incubated for 4 h at 37 °C. After incubation period, the cells were observed by confocal microscopy **(A)**, and the supernatants were used for cytotoxic activity assay **(B)** and cytokine production assay **(C)**. Scale bar, 5 µm. All experiments were performed in triplicate and data in B and C represent the mean ± SEM of independent experiments. P-values calculated by two-tailed Student’s t-test. *P<0.05; ns, not significant.

Proteome analysis revealed that *S. pyogenes* EVs enclose particular virulence-related proteins such as SLO and Nga, suggesting the involvement of EVs in the pathogenicity of *S. pyogenes*. To investigate the involvement of EVs in GAS pathogenicity, dTHP-1 cells were treated with EVs from wild-type or *slo* mutant strains, cytotoxic activity was measured, and morphological changes were observed. We first quantified the number and checked the sizes of the EVs from the *slo* mutant strains, and found no significant differences in them between the wild-type and *slo* mutants (**Supplementary Figure 5**). A large cytotoxic effect with morphological changes was observed in dTHP-1 cells with EVs from SSI-1 wild-type, but not in cells cultured with EVs from the *slo* mutant (**Figure 6A**). Furthermore, SSI-1 wild-type EVs showed 16.1% cytotoxicity in dTHP-1 cells, while this activity was significantly decreased in the *slo* mutant (mean, 3.1%) (**Figure 6B**). This result is consistent with the morphological observations, indicating that SSI-1-derived EVs induce *slo*-dependent cytotoxicity in dTHP-1 cells. Conversely, JRS4 wild-type EVs showed fewer morphological changes and less cytotoxic activity (mean, 3.9%) in dTHP-1 cells than the SSI-1 wild-type EVs. Furthermore, the cytotoxicity profile for JRS4 ∆*slo* EVs was similar to that of wild-type JRS4. In the cytokine profile obtained from the ELISA assay, both SSI-1 and JRS4 ∆*slo* EVs had similar detection levels to those of the wild-type strains for TNF-α, but IL-8 production only increased in the JRS4 ∆*slo* EVs (**Figure 6C**). This suggests that the IL-8 synthesis pathway or IL-8 secretion in the cells treated with SSI-1 EVs was suppressed in a SLO-independent manner. In contrast, compared with those in the wild-type EVs, IL-1 beta production decreased in the cells treated with EVs from both SSI-1 and JRS4 ∆*slo*, which indicates that SLO-dependent inhibition of the IL-1 beta synthesis pathway or its secretion occurred.

## Discussion

Bacterial EVs are an important tool for delivering bioactive cargo such as proteins, nucleic acids, and other biomolecules to host cells, inducing pathogenicity and modulation of host innate immune responses. (Yanez-Mo et al., 2015; Toyofuku et al., 2019). In this report, we performed a comprehensive proteome analysis of EVs from invasive and non-invasive GAS strains and show that these protein cargos are involved in the induction of both cytotoxicity and inflammatory responses, leading to the pathogenicity of GAS.

One important finding is that the invasive strain SSI-1 produced larger quantities of EVs than the non-invasive strain JRS4. The number of EVs is partly controlled by the CovRS two-component system and disruption of genes involved in the system increases EV production (Resch et al., 2016). Interestingly the invasive strain SSI-1 has non-synonymous mutations in *covRS* that are not present in JRS4 (Uno et al., 1992; Roobthaisong et al., 2017). These findings are consistent with our results showing higher EV production in invasive strains, supporting the possibility that EVs can contribute to the pathogenicity of GAS. Our proteome profiling also revealed that various proteins are enclosed in GAS EVs, as was previously reported (Resch et al., 2016). For example, D-alanyl-lipoteichoic acid biosynthesis protein (DltD), which is involved in the d-alanylation of lipoteichoic acid (LTA) by transferring d-alanine to teichoic acids on the cell surface, was present in EVs from the two GAS strains we studied and was enriched in the JRS4 EVs. It has been shown that the vesicle-mediated DltD protein contributes to the formation of d-alanylated LTA in *S. aureus* (Wood et al., 2018). This implies that GAS EVs could also play a role in compensating for the molecules required for their biological processes in the population. Additionally, our proteomic data revealed the presence of several proteins related to bacterial efflux pumps (ABC and RND-type transporters). These transporters are widely found in proteomics analyses of EVs from other bacterial species (Gurung et al., 2011; Wagner et al., 2018), and may actively help to export the virulence-related proteins and enzymes enclosed in EVs. Alternatively, these transporters on EVs might be one of the candidate protein types for bacteriophage binding, as a previous study on the inhibition of bacteriophage infection by *Vibrio cholerae* EVs has shown (Reyes-Robles et al., 2018). Thus, GAS may take advantage of EV-mediated proteins in order to modulate their surrounding environments or evade phage infection of cells as a decoy, thereby contributing to bacterial survival or adaptation in various environments. Additionally, we identified many GAS virulence-related proteins in the cargo of GAS EVs, but their presence and abundances differed between the invasive and non-invasive strains. These differences might be related to the mechanism underlying EV production in the two strains. Alternatively, the different cell-surface components may be involved in the selection of the EV cargo in GAS. Notably, two proteins, SLO and Nga, are associated with inhibition of lysosomal fusion with GAS-containing autophagosomes to form functional degradative autolysosomes, thereby contributing to the protection of GAS *via* xenophagic killing (O’seaghdha and Wessels, 2013; Toh et al., 2019). GAS EVs may also be involved in the enhancement of GAS intracellular survival during or prior to infection.

In this study, we used two strains, SSI-1 (serotype M3) and JRS4 (serotype M6), as representative invasive and non-invasive strains, respectively, although there are other M types that cause invasive GAS disease such as M1 and M12. Furthermore, recent epidemiological evidence has revealed the rapid emergence of the GAS genotype *emm89* as a leading cause of disease worldwide (Shea et al., 2011; Turner et al., 2015). Therefore, we also need to consider performing further studies to compare EVs from invasive and non-invasive types using additional M-type strains.

Another important finding in this study is that EVs from the invasive strain exhibited more severe effects on the human cells than the non-invasive strain. In fact, SSI-1 has less efficiency for adhesion to and invasion of host cells compared with JRS4 (Roobthaisong et al., 2017). This might be due to the strong hyaluronic acid capsule produced by SSI-1, which prevented their own penetration into the cells. Therefore, we hypothesized that EVs from SSI-1 and JRS4 play different roles in the GAS infection strategy and pathogenicity. Our results demonstrated that SSI-1 EVs induced cytotoxicity in THP-1 cells and caused an inflammatory response, indicating that EVs could be the main contributors to biological effects in host cells rather than the invading bacterial cells being primarily responsible. In gram-positive bacteria, proteins are generally secreted by translocation across a single membrane *via* the Sec pathway or the Tat (two-arginine) pathway (Tseng et al., 2009). Together with these general secretion pathways, bacterial EVs represent an alternative mechanism to release proteins with or without signal peptides into the extracellular space. Furthermore, EVs are stable in biological fluids and contribute to the preservation of proteins against degradation. The vacuolating VacA toxin is a major virulence factor in *Helicobacter pylori*, where it is closely associated with membrane vesicles (Keenan et al., 2000). VacA is stable in eukaryotic cells, and its toxicity can be induced in the days following internalization (Sommi et al., 1998). GAS may also deliver virulence-related proteins in mediating EVs, while keeping the host cells stable in terms of activity and structure, because SSI-1 EVs showed *slo*-dependent cytotoxic activity. Conversely, JRS4, which is capable of cell invasion despite causing non-invasive disease, may use EVs as a means to prevent antibacterial mechanisms such as autophagy, thereby leading to enhancement of their own survival in the intracellular environment after infection.

The innate immune system is an important frontline defense against invading and resident bacteria. Bacterial products, such as flagellin, peptidoglycan, lipopolysaccharide, and DNA are detected by Toll-like receptors and NOD-like receptors in host cells. Stimulation by them leads to the recruitment of adaptor proteins and downstream activation of transcription factors *via* various pathways, which leads to pro-inflammatory cytokine (IL-6, IL-8, TNF-α) production and NLRP3 inflammasome activation (IL-1β, IL-18) (Ellis and Kuehn, 2010; Kaparakis-Liaskos and Ferrero, 2015; Pathirana and Kaparakis-Liaskos, 2016). Thus, bacterial EVs are strongly associated with host inflammatory responses, and a number of studies have demonstrated their importance in bacterial EV–host interactions. However, knowledge about gram-positive bacterial EVs is limited. In this study, we observed that GAS EVs also induce the transcriptional expression of IL-8, TNF-α, and IL-1β. Interestingly, SSI-1 WT-derived EVs were able to induce IL-8 expression at the transcriptional level; however, we could not detect this cytokine by ELISA. Additionally, we found that IL-8 production increased in the JRS4 ∆*slo*-derived EVs, whereas this cytokine was not detected in the cells treated with SSI-1 ∆*slo*-derived EVs as WT EVs. These results suggest that SSI-1 EVs may suppress IL-8 production or its secretion in an SLO-independent manner. C5a peptidase, a well-studied endopeptidase in GAS and one of the proteins identified in the proteomic profile of EVs from the SSI-1 strain (**Supplementary Table 2**), is capable of degrading and functionally inactivating IL-8, thereby contributing to the establishment of local infections (Cleary et al., 1992; Edwards et al., 2005; Zinkernagel et al., 2008). This peptidase (or other molecules) is transferred into host cells by EVs where it might then be involved in diminishing IL-8. This mechanism for suppressing particular cytokines could also be used for enhancing the ability of GAS to evade the innate immune response prior to or subsequent to their infection. However, current data do not clearly show how EVs from SSI-1 diminished the activity or secretion of IL-8 in host cells; therefore, further investigation is required to reveal the detailed mechanism. Nevertheless, we found that IL-1β secretion decreased significantly in an SLO-dependent manner. It has been reported that SLO is required for IL-1β secretion and for stimulating the NLRP3 inflammasome (Harder et al., 2009). Our results suggest that EV-mediated SLO is also associated with this mechanism. Thus, GAS EVs also contribute to modulation of host inflammatory responses in various ways. Our previous work demonstrated that the inflammatory response is attenuated by disruption of the Golgi complex and the post-Golgi secretory pathway during the GAS invasion process (Nozawa et al., 2021). Hence, the results of the present study imply that attenuation of the inflammatory response is triggered by living bacterial cells and bacterial component-containing EVs. Although further studies are needed to confirm our hypothesis, these findings suggest that EVs play an important role in intracellular survival, and this leads to the establishment of a GAS infection.

While bacterial EVs contribute to host pathogenicity, EVs are also expected to be potential vaccine candidates against bacterial infection (Collins, 2011; Acevedo et al., 2014). Neisseria meningitis, the bacterium responsible for invasive meningococcal disease, is one of the first successful cases for the application of EVs as an effective vaccine candidate (Granoff, 2010; Andrews and Pollard, 2014; Christodoulides and Heckels, 2017). However, a safe and effective human GAS vaccine is not yet available because a number of obstacles hinder the development of such a vaccine, including serotype diversity, antigenic variation, and pronounced differences in the geographical distribution of serotypes (Steer et al., 2009; Mcmillan et al., 2013; Walker et al., 2014). To address this issue, Mark et al. reported several antigen candidates identified by large-scale comparative genome analysis (Davies et al., 2019). Thus, our findings reported in this study will help us to not only understand the GAS virulence mechanism but also contribute to the development of an effective vaccine against GAS infection.

## Data Availability Statement

The datasets presented in this study can be found in online repositories. The names of the repository/repositories and accession number(s) can be found in the article/**Supplementary Material**.

## Author Contributions

KM and IN conceived and designed the study. KM, TK, AN, KS, CA, and TN contributed to the acquisition, analysis, or interpretation of the data. KM, TK, and IN contributed to the writing of manuscript and the preparation of figures. All authors contributed to the article and approved the submitted version.

## Funding

This work was supported in part by Grants-in-Aid for Scientific Research (20K07495, 20H05504, 19H03471, 19K07537, 18K07109) from the Japan Society for the Promotion of Science, the Joint Research Project of the Institute of Medical Science, the University of Tokyo, and the Research Program on Emerging and Re-emerging Infectious Diseases (20fk0108073h0003 and 20fk0108130h0401) and J-PRIDE (19fm0208030h0003) from the Japan Agency for Medical Research and Development (AMED).

## Conflict of Interest

The authors declare that the research was conducted in the absence of any commercial or financial relationships that could be construed as a potential conflict of interest.
