# Supplementary material for: Biological Effect of Streptococcus pyogenes-Released Extracellular Vesicles on Human Monocytic Cells, Induction of Cytotoxicity, and Inflammatory Response
Source: Front Cell Infect Microbiol. 2021 Jul 19;11:711144. doi: 10.3389/fcimb.2021.711144 (PMC8326760; doi:10.3389/fcimb.2021.711144)
Supplement: Supplementary file 1 [file DataSheet_1.pdf]

Supplementary Material

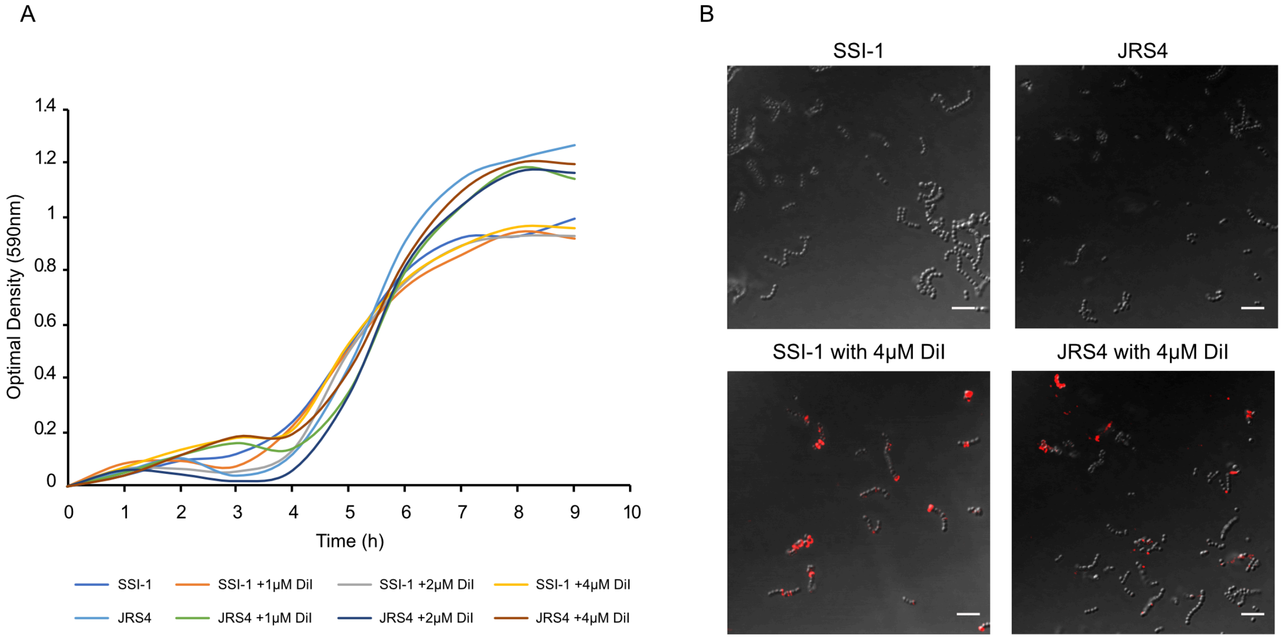

Supplementary Figure 1. Growth of DiI-labelled *S. pyogenes* cells. Two *S. pyogenes* strains, SSI-1 and JRS4, were grown at 37ºC in THY medium, supplemented with final concentrations of 1, 2, 4μM DiI solution. Growth of bacterial cells were measured at OD590 and shown in growth curve (A). The grown bacterial cells labelled with DiI (red) were further observed by fluorescence microscopy (B). Scale bar, 5μm.

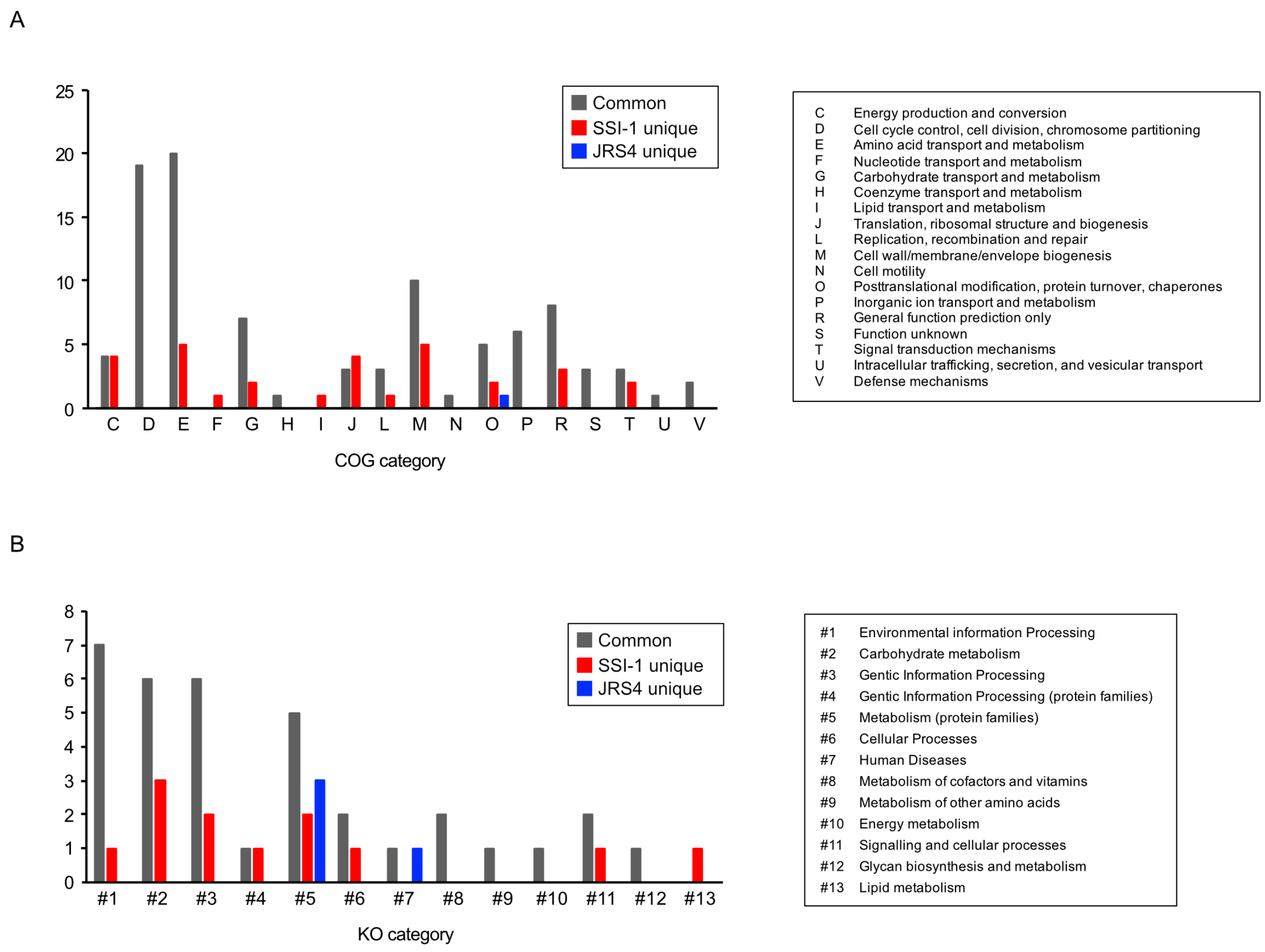

Supplementary Figure 2. COG and KO classification of proteins identified in EVs from SSI-1 and JRS4. Common or strain-specific proteins identified in EVs from SSI-1 and JRS4 were analyzed for COG (A) or KO classification (B). COGs were predicted by searching the amino acid sequences of the genes against COG hidden Markov models downloaded from the eggNOG 4.5 database. Protein functions were further assigned using BlastKOALA tool via KEGG database.

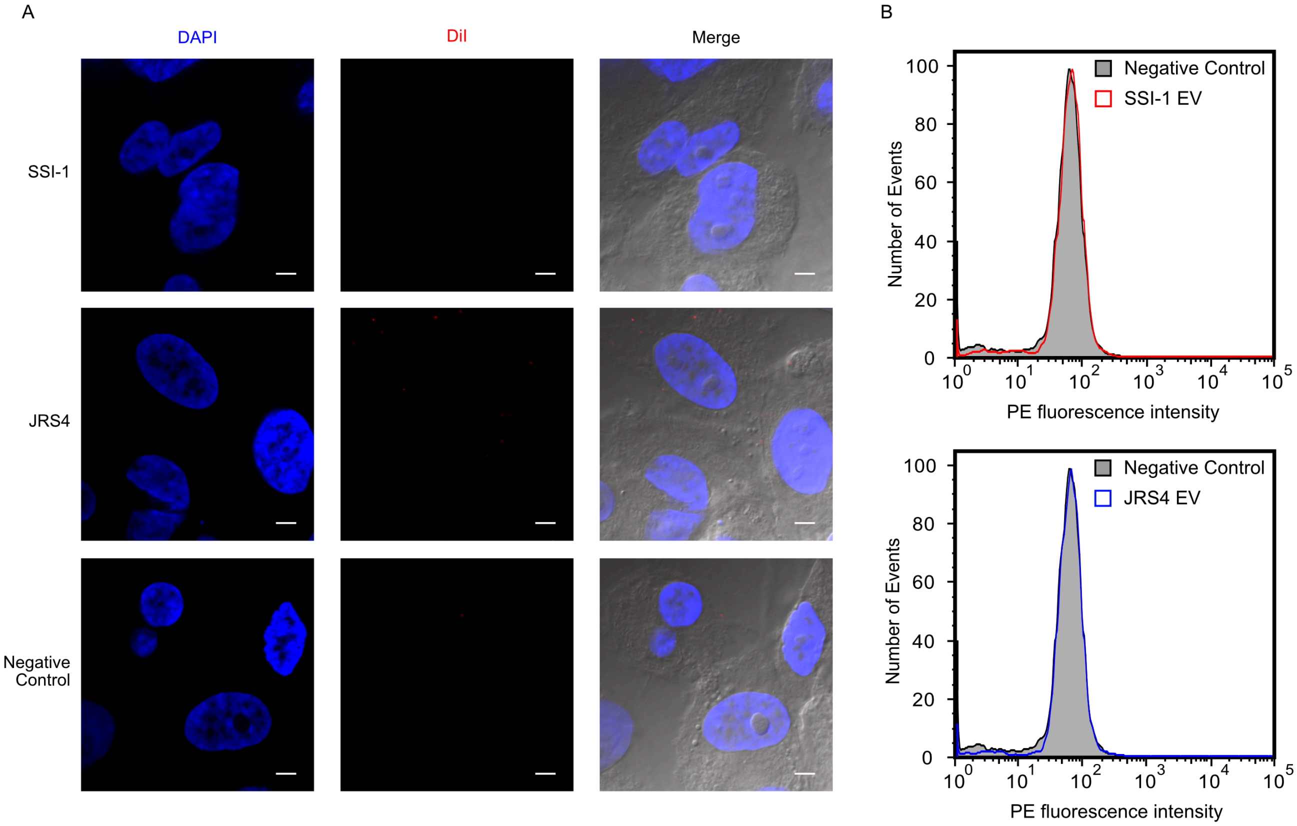

Supplementary Figure 3. Uptake of DiI-labelled EVs and the efficiency in dTHP-1 cells incubated at 4ºC. A) The dTHP-1 cells were co-incubated with 10 µg of DiI-labled EVs (red) solution for 2 h at 4 ºC and observed by confocal fluorescence microscopy. Cellular DNA was stained with DAPI (blue). Scale bar, 5 µm. B) Flow cytometric analysis of the fluorescence transferred by EVs into dTHP-1 cells. The staining of directly labelled cells and the negative control (cells treated with 4µM DiI solution) are shown as overlays. The PE fluorescence intensity was measured after 2 h incubation period.

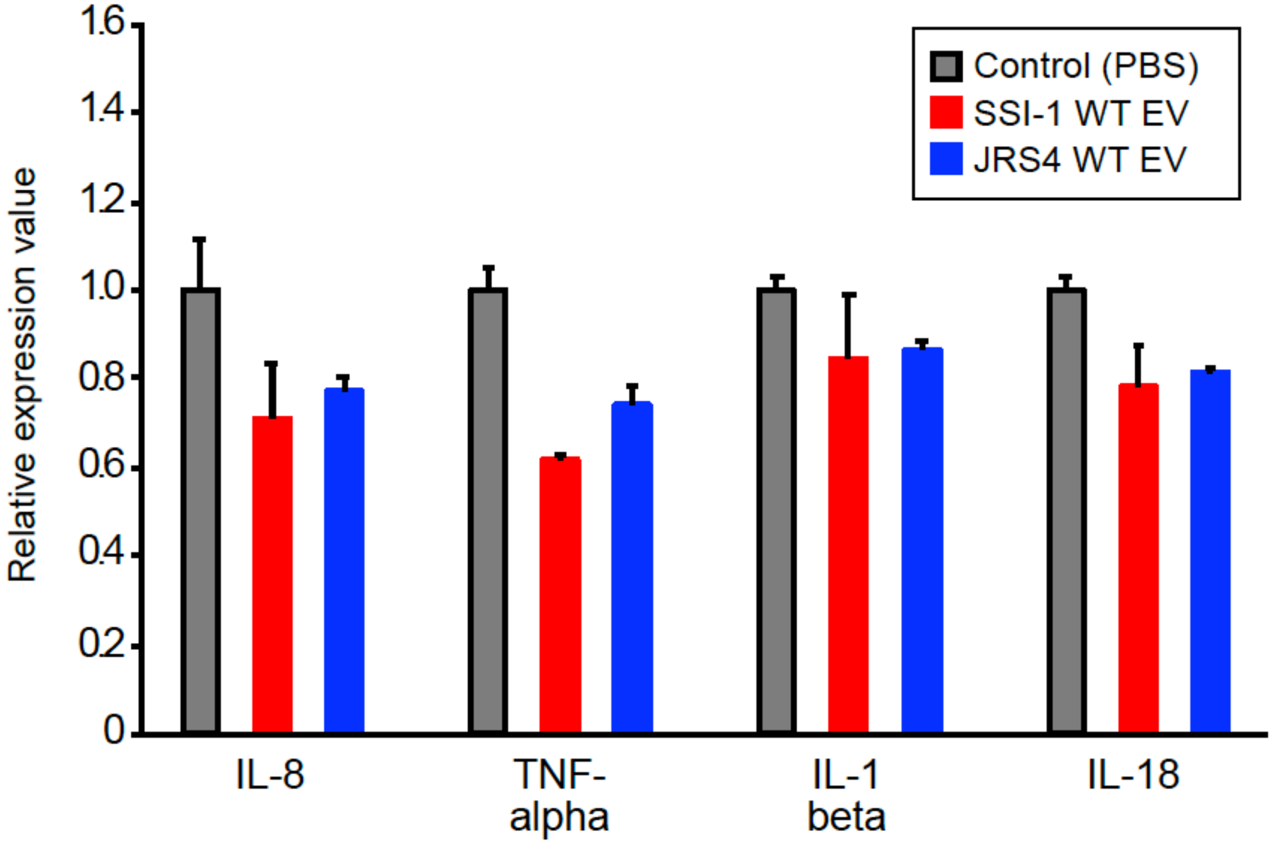

Supplementary Figure 4. Transcriptional level of cytokine expressions at 4ºC. 10 µg of EVs solution was treated with dTHP-1 cells and incubated for 4 h at 4ºC. The transcriptional level of IL-8, TNF-α, IL-1 beta, and IL-18 were examined using real-time PCR assay. Data represent the mean ± SEM of independent experiments.

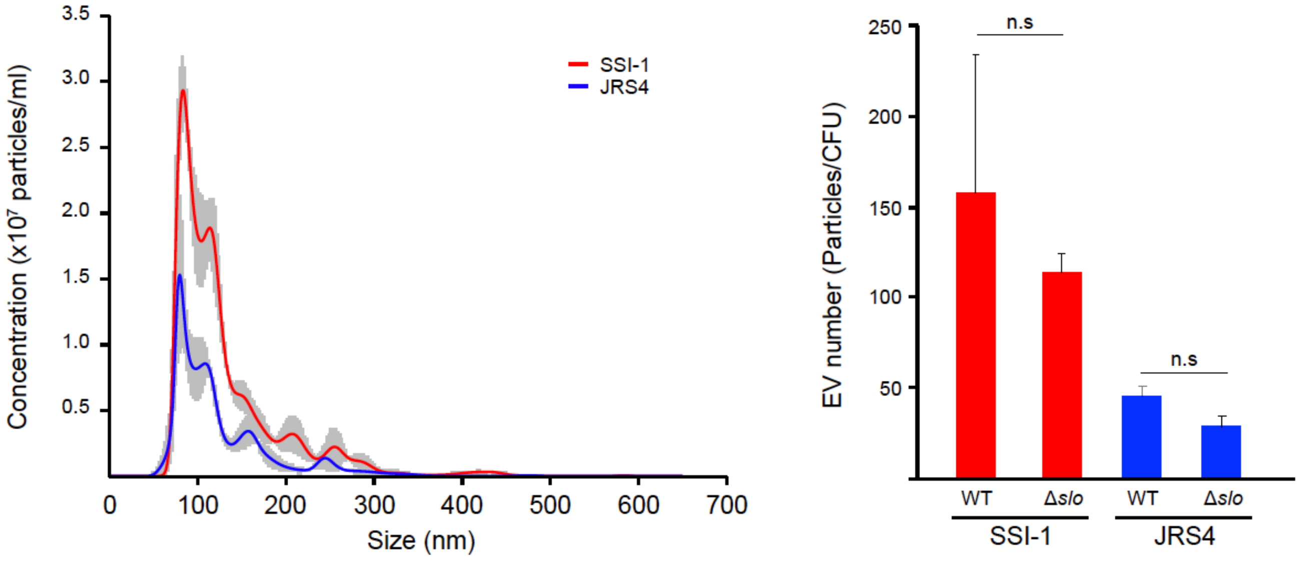

Supplementary Figure 5. Quantification and size distribution of the EVs released from the *slo* mutants of *S. pyogenes* SSI-1 and JRS4. Quantification and size distribution of *S. pyogenes* Δ*slo* EVs were analyzed by NanoSight (left-hand panel), and EV numbers (particles/CFU) were calculated from NanoSight data and the total cells in colony forming unit (CFU)/ml (right-hand panel). All experiments were performed in duplicate. Data represent the mean ± SEM of independent experiments.
